# uPAR antibody (huATN-658) and Zometa reduce breast cancer growth and skeletal lesions

**DOI:** 10.1038/s41413-020-0094-3

**Published:** 2020-04-17

**Authors:** Niaz Mahmood, Ani Arakelian, Haseeb Ahmed Khan, Imrana Tanvir, Andrew P. Mazar, Shafaat A. Rabbani

**Affiliations:** 1 Department of Medicine, McGill University, Montréal, QC H4A3J1 Canada; 2 Fatima Memorial Hospital System, Lahore, Pakistan; 3 Monopar Therapeutics, Wilmette, IL 60091 USA

**Keywords:** Cancer, Bone cancer

## Abstract

Urokinase plasminogen activator receptor (uPAR) is implicated in tumor growth and metastasis due to its ability to activate latent growth factors, proteases, and different oncogenic signaling pathways upon binding to different ligands. Elevated uPAR expression is correlated with the increased aggressiveness of cancer cells, which led to its credentialing as an attractive diagnostic and therapeutic target in advanced solid cancer. Here, we examine the antitumor effects of a humanized anti-uPAR antibody (huATN-658) alone and in combination with the approved bisphosphonate Zometa (Zoledronic acid) on skeletal lesion through a series of studies in vitro and in vivo. Treatment with huATN-658 or Zometa alone significantly decreased human MDA-MB-231 cell proliferation and invasion in vitro, effects which were more pronounced when huATN-658 was combined with Zometa. In vivo studies demonstrated that huATN-658 treatment significantly reduced MDA-MB-231 primary tumor growth compared with controls. In a model of breast tumor-induced bone disease, huATN-658 and Zometa were equally effective in reducing skeletal lesions. The skeletal lesions were significantly reduced in animals receiving the combination of huATN-658 + Zometa compared with monotherapy treatment. These effects were due to a significant decrease in osteoclastic activity and tumor cell proliferation in the combination treatment group. Transcriptome analysis revealed that combination treatment significantly changes the expression of genes from signaling pathways implicated in tumor progression and bone remodeling. Results from these studies provide a rationale for the continued development of huATN-658 as a monotherapy and in combination with currently approved agents such as Zometa in patients with metastatic breast cancer.

## Introduction

Despite the advances made in the field of cancer therapeutics, treatment of metastatic disease remains a challenge and accounts for more than 90% of the breast cancer-related deaths worldwide.^1^ The median survival for patients with metastatic or late-stage breast cancer is between 2 and 4 years.^2^ Even though less than 10% of the newly diagnosed cases of breast cancer patients present with metastasis, 30%–50% of patients diagnosed with early-stage breast cancer eventually develop metastasis despite undergoing standard of care anticancer therapeutic regimens.^3,4^ In addition, increased numbers of patients develop treatment-resistant metastatic breast cancer due to the vigorous use of cytotoxic agents.^4^

Bone is a common site for breast cancer metastasis, which can cause various complications, including hypercalcemia, increased fragility fractures, intractable bone pain, and nerve compression.^5,6^ These complications are collectively known as skeletal-related events, and have a profound effect on decreasing the overall quality of life (QOL) and contribute significantly to morbidity and mortality.^7^ In order to improve patient outcome and QOL for survivors, there is an urgent need for the development and validation of rational and effective anticancer therapeutic strategies that can be effective in reducing breast tumor growth in nonskeletal and skeletal sites. The uPA–uPAR axis is a central player in mediating metastatic progression and is frequently overexpressed in different cancers.^8^ The proteolytic effects of urokinase plasminogen activator (uPA) are localized within the tumor cell environment by its receptor uPAR, which also triggers the activation of several key oncogenic signaling pathways independent of proteolysis.^9,10^ Early anticancer strategies were directed towards interfering with the binding of uPA to its receptor uPAR.^11–13^ However, these approaches showed modest anticancer effects, possibly because they were based on the previously accepted notion that uPA is the major ligand of uPAR. Other studies have demonstrated that uPAR also binds to integrins and vitronectin, and thereby plays a significant role in the regulation of angiogenesis and activation of integrins/vitronectin activated pathways within the tumor microenvironment.^14–16^ Moreover, loss-of-function studies have revealed that attenuation of uPAR expression causes a substantial reduction of tumor cell growth and metastasis.^17–20^ Taken together, these studies provide convincing evidence for the role of uPAR as a central player in the uPA–uPAR axis and credential uPAR as a therapeutic target for the treatment of advanced solid cancer, including breast cancer. We have focused on developing an antibody-based therapeutic strategy targeting uPAR. From a panel of anti-uPAR clones, we selected ATN-658 for further development based on demonstrating its ability to block multiple oncogenic signaling pathways, tumor growth, and metastasis in several xenograft tumor models^21–23^ ATN-658 was subsequently humanized (huATN-658; MNPR-101) and is now in late preclinical development. The huATN-658 antibody binds to the DIII domain of uPAR near its C-terminal and does not interfere with uPA or vitronectin binding to uPAR.^24^ Instead, huATN-658 blocks uPAR-integrin interactions.^24^ The fact that huATN-658 can bind to and neutralize uPAR functions regardless of its uPA binding status makes it pharmacologically attractive since it does not require overcoming the barrier associated with the displacement of endogenous uPA that is already present in a complex with uPAR.^22^

In the current study, we assessed whether targeting uPAR is a viable anticancer strategy against breast tumor growth using a xenograft model. Subsequently, we examined the effects of huATN-658 alone and in combination with the approved amino-bisphosphonate Zometa on skeletal lesion using our well-established intratibial model of breast cancer. Our data show that huATN-658 significantly reduced tumor volume and skeletal lesion. Furthermore, the combination of huATN-658 + Zometa not only reduced tumor burden but also resulted in blocking the development of skeletal lesions in most animals as compared with vehicle or monotherapy controls.

## Results

### uPAR-encoding *PLAUR* gene is upregulated in breast cancer and associated with aggressiveness of the disease

**Fig. 1:**
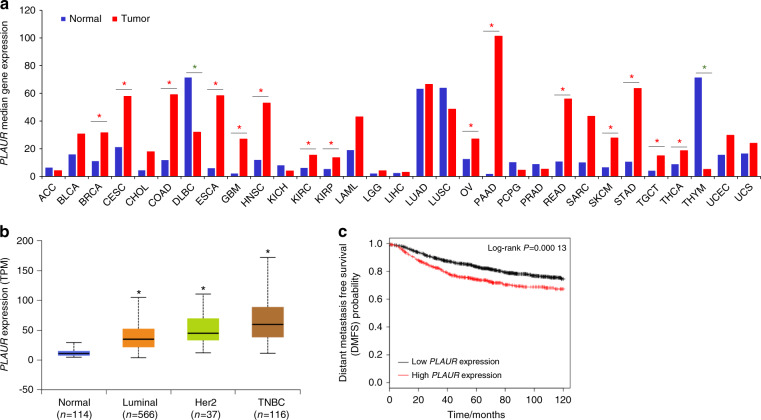
*PLAUR* is upregulated in breast and other cancers. **a** The median expression of the human *PLAUR* gene in normal and tumor tissues of 31 different types of cancer. Cancer abbreviations: ACC adrenocortical carcinoma, BLCA bladder urothelial carcinoma, BRCA breast invasive carcinoma, CESC cervical squamous cell carcinoma and endocervical adenocarcinoma, CHOL cholangio carcinoma, COAD colon adenocarcinoma, DLBC lymphoid neoplasm diffuse large B-cell lymphoma, ESCA esophageal carcinoma, GBM glioblastoma multiforme, HNSC head and neck squamous cell carcinoma, KICH kidney chromophobe, KIRC kidney renal clear cell carcinoma, KIRP kidney renal papillary cell carcinoma, LAML acute myeloid leukemia, LGG brain lower grade glioma, LIHC liver hepatocellular carcinoma, LUAD lung adenocarcinoma, LUSC lung squamous cell carcinoma, OV ovarian serous cystadenocarcinoma, PAAD pancreatic adenocarcinoma, PCPG pheochromocytoma and paraganglioma, PRAD prostate adenocarcinoma, READ rectum adenocarcinoma, SARC sarcoma, SKCM skin cutaneous melanoma, STAD stomach adenocarcinoma, TGCT testicular germ cell tumors, THCA thyroid carcinoma, THYM thymoma, UCEC uterine corpus endometrial carcinoma, UCS uterine carcinosarcoma. The cancer types where *PLAUR* expression is significantly upregulated and downregulated in tumor tissues relative to adjacent normal tissues are marked by ‘red’ and ‘green’ asterisks, respectively. **b** The *PLAUR* expression is elevated in all subtypes of breast cancer. Significant differences are represented by asterisks (*P < 0.05). **c** Kaplan–Meier survival curve using gene expression datasets of 1 746 breast cancer patients shows that higher expression of the *PLAUR* gene is associated with a significantly poor distant metastasis-free survival (DMFS). The microarray-based gene expression values from the 1 764 patients were divided into two groups based on the median expression of the *PLAUR* gene. In this case, the cutoff value was 282 (automatically done by the KM-plotter algorithm) while the expression range of the probe was between 23 and 1 191. Based on this cutoff value, *PLAUR* gene expressions in 1 301 out of the 1 746 patients were analyzed in the low expression group while expression values from 445 patients were analyzed in the high expression group during DMFS analysis

Elevated uPAR (*PLAUR)* expression has been typically linked with the aggressiveness of breast cancer.^25^ Many of these studies were done before the large-scale transcriptomic datasets derived from cancer patients were available. Therefore, we first interrogated the publicly available RNA-Sequencing datasets of The Cancer Genome Atlas (TCGA) and found that *PLAUR* expression is elevated in breast and many other common cancers when compared with their respective control tissues (Fig. 1a, Supplementary File 1, Fig. S1). Interestingly, the *PLAUR* gene expression in tumor tissues showed a statistically significant increase in ~48% (15 out of the 31) of the cancer types tested, while a substantial downregulation was detected in only ~6% (2 out of 31). A similar trend in the elevated expression of the *PLAUR* gene was observed upon the analysis of different cancer types in the Oncomine database where the highest expression is seen in breast tumors relative to normal tissues (Supplementary File 1, Fig. S2). PLAUR expression was increased in the breast cancer subtypes that have historically demonstrated the shortest median time to recurrence, where the highest expression is seen in the triple-negative subtype (Fig. 1b). It should be noted that these sample sets only look at *PLAUR* gene expression and not uPAR protein and that they have been obtained from bulk tumor extraction mostly of primary and not metastatic breast tumors. *PLAUR* is not uniformly expressed in tumors and its expression may occur only in a subset of tumor-associated cells.^26^ These caveats have been observed in other cancer types, which sometimes create a disconnect between gene expression and protein expression (e.g., immunohistochemistry) data in the same cancer type.^27^ Nevertheless, regardless of how *PLAUR* or uPAR expression is assessed, the differential expression between tumor and normal tissue is clearly evident.

We then investigated the prognostic significance of *PLAUR* using the (Kaplan–Meier plotter) KM-plotter dataset and found that increased *PLAUR* expression correlates with significantly poor 10-year (120 months) distant metastasis-free survival in patients with breast cancer [log-rank *P* = 0.000 13; at 5% false discovery rate threshold] (Fig. 1c). Furthermore, the median survival of the breast cancer patients with low *PLAUR* gene expression is 118.62 months, which decreases to 56.91 months in the group with high expression of the gene. Taken together, our analysis of different publicly available breast cancer datasets independently demonstrates that higher *PLAUR* expression is associated with increased aggressiveness of breast cancer and that all types of breast cancer express higher levels of uPAR than normal breast tissue.

### huATN-658 attenuates mammary tumor growth in vivo

**Fig. 2:**
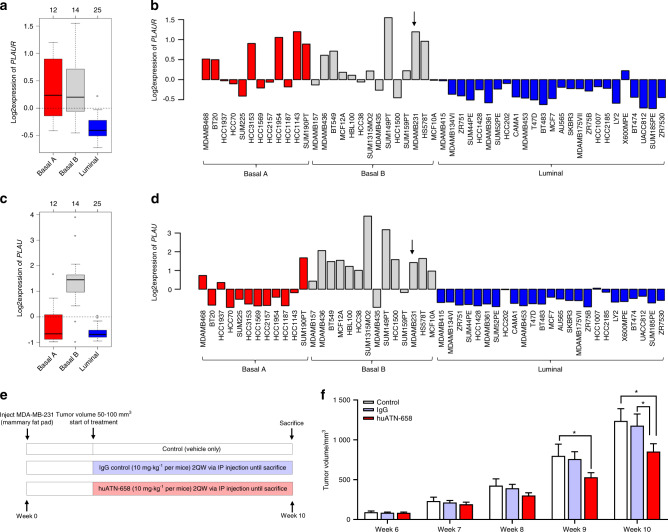
Effect of huATN-658 on the development of MDA-MB-231 mammary fat pad tumors. Box plot showing the expression of *PLAUR* and its ligand *PLAU* in cell lines (**a**, **c**). *PLAUR* and *PLAU* gene expression in 51 different cell lines (**b**, **d**)**. e** Schematic of the protocol used for huATN-658 treatment in an orthotopic model of breast cancer. Female CD-1 nude mice were inoculated with 5 × 10^5^ MDA-MB-231 cells. Once the tumor volume reached between 50 and 100 mm^3^, mice were treated with control IgG (control), 10 mg·kg^−1^ human IgG or huATN-658 twice a week via the intraperitoneal route for 5 weeks. **f** Tumor volume was determined at weekly intervals. Results are shown as the mean ± SEM (*n* = 9 animals/group). Significant differences were measured using ANOVA followed by post hoc Tukey’s test and are represented by asterisks (*P < 0.05)

We next aimed to evaluate the antitumor potential of the huATN-658 antibody targeting the uPAR protein in a xenograft model of breast cancer. Even though the genes (*PLAUR* and *PLAU*) encoding for uPAR and uPA proteins show an overall higher expression in the basal B triple-negative subtypes (Fig. 2a, c), the levels of detectable *PLAUR* and *PLAU* varies between different cell lines within the same subtype (Fig. 2b, c). Since human MDA-MB-231 cells express relatively high levels of *PLAUR* (Fig. 2b) and its ligand encoding gene *PLAU* (Fig. 2d), we chose to use this cell line for all of our in vivo and in vitro studies.

Female CD-1 nude mice were inoculated with 5 × 10^5^ MDA-MB-231 human breast cancer cells orthotopically into the fourth mammary fat pad as previously described.^28^ The mice were assessed for the presence of palpable tumors at weekly intervals. Once the mammary tumor volume reached between ~50 and 100 mm^3^, they were randomized into three different groups and treated with vehicle alone, 10 mg·kg^−1^ control IgG or huATN-658 antibody twice a week via intraperitoneal route for 5 weeks (week 6 to week 10 post tumor cell inoculation) (Fig. 2e). Control group of animals treated with vehicle alone or control IgG showed a progressive increase in primary mammary tumor volume throughout the course of these studies, and there was no significant difference in tumor volume between these two control groups. In contrast, the experimental group of animals receiving huATN-658 showed a significant decrease in mammary tumor volume compared with both control groups (Fig. 2f). No physical or behavioral abnormalities were noted in the huATN-658-treated animals during this study, consistent with previously published studies using huATN-658.^22–24^

### huATN-658 and Zometa combination decreases cell proliferation and invasion in vitro

**Fig. 3:**
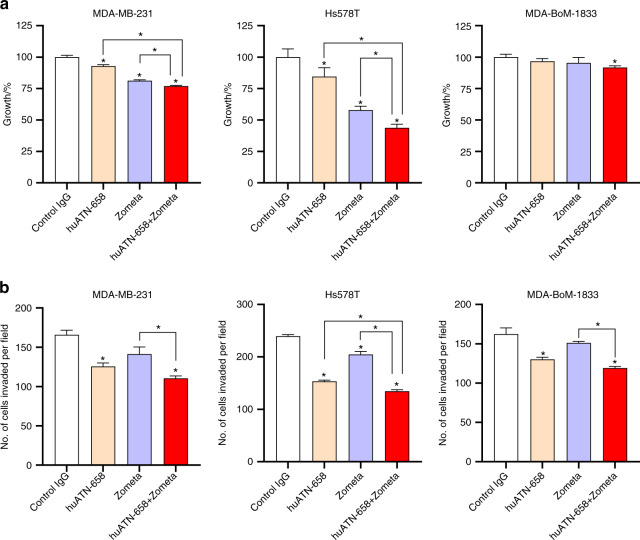
Effect of huATN-658, Zometa, and their combination in vitro. **a** Human MDA-MB-231, Hs578T, and MDA-BoM-1833 cells were plated and treated with control IgG alone, huATN-658 (50 μg·mL^−1^), Zometa (10 μmol·L^−1^), and a combination of huATN-658 and Zometa. Cells were counted after 2 days (48 h) post treatment using a Coulter counter. Results are shown from two different experiments done in duplicates. **b** The invasive capacity of the cells was evaluated using a Boyden chamber matrigel invasion assay of control and treated cells. After incubation for 18 h, cells were fixed, stained, and randomly selected fields were counted. Results are shown as the mean ± SEM from two different experiments done in duplicates. The statistically significant differences were determined using ANOVA followed by post hoc Tukey’s test and are represented by asterisks (*P < 0.05)

Since huATN-658 inhibits tumor growth while Zometa is approved for the treatment of bone metastases, we hypothesized that a combination of huATN-658 and Zometa would be more effective in blocking or reducing skeletal tumor burden as compared with huATN-658 or Zometa alone. We first evaluated the effect of the huATN-658 and Zometa combination on a panel of human breast cancer cell lines (MDA-MB-231, Hs578T, MDA-BoM-1833) in vitro. We and others have previously established the doses and schedules of huATN-658 and Zometa treatment.^22,29,30^ We found that the optimal anticancer effect is obtained following combination treatment using 50 μg·mL^−1^ huATN-658 and 10 μmol·L^−1^ Zometa for 48 h (data not shown). Treatment with huATN-658 and Zometa alone caused a significant reduction in tumor cell proliferation as compared with the control IgG-treated MDA-MB-231 and Hs578T cells, but not for the MDA-BoM-1833 cells (Fig. 3a). However, these effects were significantly more pronounced upon huATN-658 + Zometa combination treatment in all three cell lines. We also determined the coefficient of drug interaction (CDI) to assess whether the interaction between the two agents in huATN-658 + Zometa combination is synergistic, additive or antagonistic; and found an additive effect of the combination (CDI = 1.02, 0.89, and 0.99 for MDA-MB-231, Hs578T, and MDA-BoM-1833 cells, respectively) in reducing tumor cell growth at the doses used in this study.

Using a Boyden chamber Matrigel invasion assay, we next assessed the changes in the invasive properties of these cell lines treated with huATN-658, Zometa, and their combination. As shown in Fig. 3b, treatments with huATN-658 monotherapy and huATN-658 + Zometa combination caused a statistically significant reduction in invasion as compared with the cells treated with control IgG in case of all three cell lines. Zometa treatment significantly reduced the invasiveness of the Hs578T cells only but not for MDA-MB-231 and its bone metastatic variant MDA-BoM-1833. Moreover, there was a statistically significant reduction in the number of invaded cells in the huATN-658 + Zometa combination relative to the cells treated with Zometa alone in the case of all three cell lines tested. However, there was no significant difference in the number of invaded cells in the huATN-658 single agent and huATN-658 + Zometa combination treatment groups in MDA-MB-231 and MDA-BoM-1833 cell lines which suggest that huATN-658 primarily mediates the reduced invasiveness in these two cell lines.

### huATN-658 and Zometa combination treatment decreases skeletal lesion in vivo

**Fig. 4:**
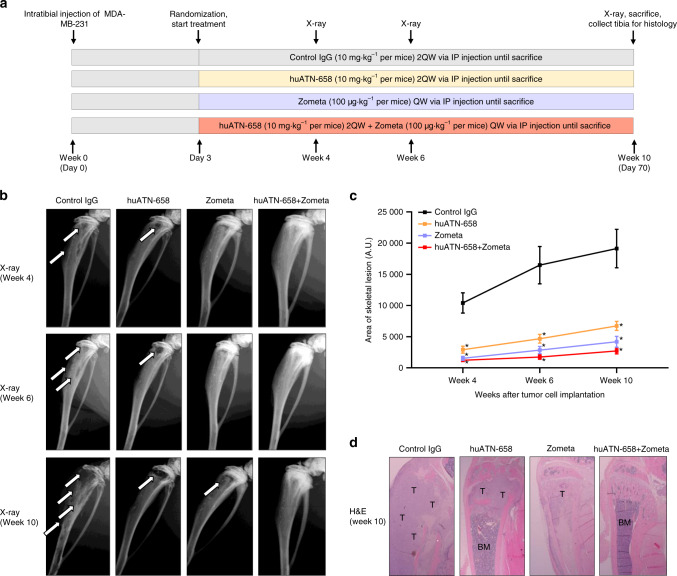
Effect of huATN-658, Zometa, and their combination on MDA-MB-231 skeletal lesions. **a** Schematic of the protocol used for treatment with huATN-658, Zometa, and their combination in an intratibial of breast cancer. The skeletal lesions were determined at weekly intervals using Kubtec digital X-ray. **b** Representative X-rays of the tibia used from each treatment group are shown where the arrows indicate the skeletal lesions. **c** Lesion area was determined by ImageJ (Fiji plugin), and the mean ± SEM of the area of skeletal lesions at different time points are plotted as a line graph. Significant differences in skeletal lesions from the control IgG animals were determined using ANOVA followed by post hoc Tukey’s test and are represented by an asterisk (*P < 0.05). **d** Histologic analysis was carried by H&E staining, and a representative bone section from each group is shown where the tumors are marked as “T” and bone marrow as “BM” (*n* = 7 animals per group)

We then moved to the main objective of the current study i.e., to examine the effect of the huATN-658 + Zometa combination on skeletal lesions using an intratibial model of breast cancer. Three days after the inoculation of 2 × 10^5^ MDA-MB-231 cells via the intratibial route, nude CD-1 nude mice were treated with control IgG alone, huATN-658 (10.0 mg·kg^−1^), Zometa (100.0 µg·kg^−1^), or huATN-658+Zometa for 10 weeks using the treatment strategy depicted in Fig. 4a. Radiologic assessment of skeletal lesions was conducted at different time intervals at weeks 4, 6, and 10 using Kubtec Digital X-ray (Fig. 4b). Control animals showed a progressive increase in skeletal lesions over time, as shown by the radiologic assessment in Fig. 4c. However, this increase was significantly less following huATN-658 and Zometa monotherapy treatment. A stabilization of skeletal lesions was seen over time in the combination huATN-658 + Zometa cohort (Fig. 4b, c). Due to the large skeletal lesions in the control group, 43% of animals died between week 6 and 9, whereas all animals treated with huATN-658, Zometa, and huATN-658 + Zometa survived until the end of the study at week 10.

Next, we sought whether these treatments have any effect on the latency to develop skeletal lesions. At week 10 post tumor cell inoculation, the percentage of animals developing skeletal lesions was 100% in control group, 57% in huATN-658, 43% in Zometa, and only 28% in the huATN-658 + Zometa-treated group of animals. At the end of the study on week 10, all animals were sacrificed, tibias were removed, decalcified and then subjected to histological analyses. The hematoxylin and eosin (H&E) staining confirmed the decrease in skeletal lesions, as seen by the X-ray analyses (Fig. 4c). While the presence of skeletal tumors was seen in the tibias of all control animals, the tumor burden was markedly decreased in all three experimental groups as determined by ImageJ (Fiji plugin) (Fig. 4d; Supplementary File 1, Fig. S3). Results from these studies showed the ability of this approach to reduce skeletal tumor burden as well as the progression of osteolytic lesions in the huATN-658 + Zometa combination group (Fig. 4b, c).

To exclude any possible cell line-specific idiosyncrasy, we also assessed the anticancer therapeutic potential of the huATN-658 + Zometa combination therapy in vivo using the bone metastatic MDA-BoM-1833 cells. Results from these studies showed that the combination-treated animals had lesser skeletal lesions compared with the control and monotherapy treated arms, results which were similar to those seen using MDA-MB-231 cells (Supplementary File 1, Fig. S4).

### huATN-658 and Zometa combination decreases the known markers for osteoclasts and tumor cell proliferation

**Fig. 5:**
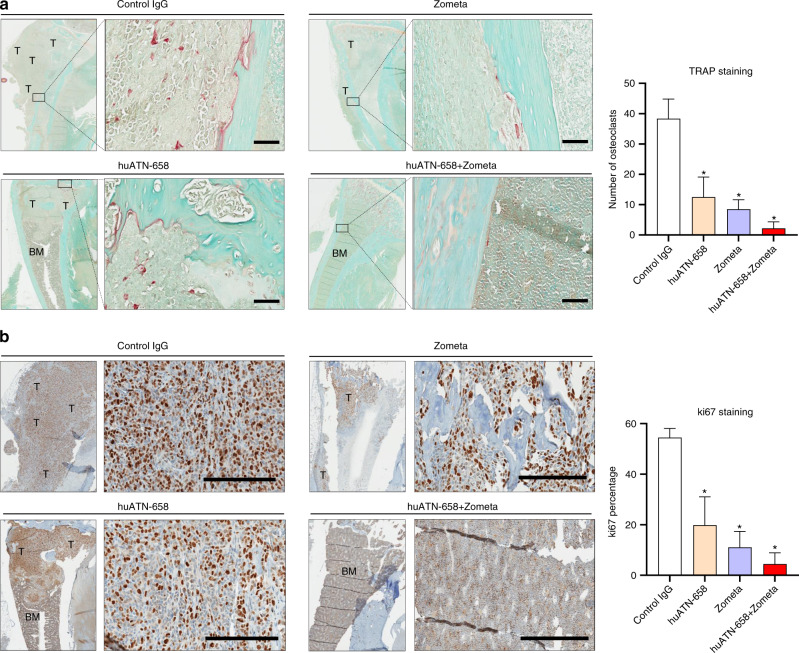
Immunohistochemical assessment of fixed tissue sections from control and treated animals. **a** The number of osteoclasts was assessed from representative tibia by TRAP staining. A lower (at ×20) and a higher magnification image (at ×400) [scale bar size = 60 μm] for the representative tibia from each group is shown on the left panel. The multinucleated osteoclast cells are stained in ‘red’. The total number of osteoclasts was counted, and the bar graph on the right panel represents the mean ± SEM of osteoclasts counted from three animals per group. **b** Tibias from different groups were subjected to immunohistochemical analysis using an antibody against the ki67 proliferation marker, as indicated by the ‘brown’ colored staining in the tissue sections from each group (left panel). The same bone sections used for TRAP assays for each groups are shown for ki67. A lower (at ×40) and a higher magnification image (at ×200) [scale bar size = 200 μm] for representative tibia from each group is shown. The bar graph in the right panel shows the mean ± SEM of the percentage of ki67 positive tumor cells from tibias from each group (*n* = 3 per group). In case of both TRAP and ki67 staining, significant differences from controls were determined using ANOVA followed by post hoc Tukey’s test and are represented by an asterisk (*P < 0.05). Here, “T” indicates tumors and “BM” indicates bone marrow

Since bone resorption mediated by osteoclasts plays a major role in the development of skeletal lesion, we next sought to determine the effects of the huATN-658 + Zometa combination on the multinucleated osteoclastic cell formation. For this, we performed TRAP staining of decalcified bone from study animals. Our data showed that huATN-658 monotherapy caused a significant decrease in the number of osteoclasts compared with the control group by 68% (Fig. 5a). As expected, Zometa, which is an approved bisphosphonate for treating breast cancer patients with bone metastasis,^31^ also caused a significant reduction in the number of osteoclasts (76%). However, these effects were most robust in the huATN-658 + Zometa combination-treated animals, with an ~95% reduction in the number of osteoclasts from the animals receiving monotherapies of huATN-658 and Zometa, respectively (Fig. 5a).

We also examined the antiproliferative effects of the different treatments by probing the fixed tibial sections from study animals using an antibody against Ki67, a cell proliferation marker. Our immunohistochemical analyses showed that the percentage of Ki67 positive cells was markedly decreased in the tissue sections from huATN-658- and Zometa-treated animals compared with the control animals (Fig. 5b). Moreover, the tissues from huATN-658 + Zometa combination-treated animals showed >90% reduction of Ki67 positive cells compared with the animals treated with control IgG (Fig. 5b).

### Gene expression changes in MDA-MB-231 transcriptome following huATN-658 + Zometa treatment

**Fig. 6:**
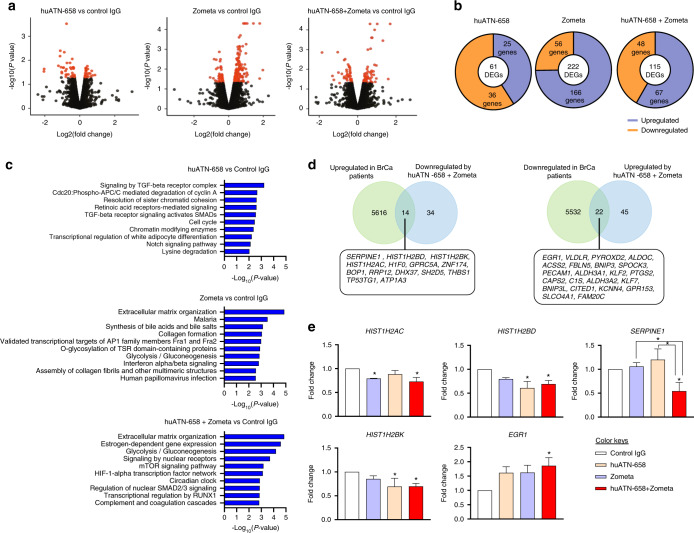
Gene expression analysis of MDA-MB-231 cells from control and different treatment groups by RNA-Sequencing. **a** Volcano plot of the significantly differentially expressed genes are shown (*n* = 3 per group). Extreme nonsignificant values were filtered out before generating the volcano plot for better representation of significantly differentially expressed genes. **b** Donut plot of DEGs in each treatment group compared with controls. **c** Functional pathway analysis (from KEGG, PID, and Reactome database) of the DEGs in different treatment groups. The top ten pathways in each treatment group are shown. **d** Venn diagram up- and downregulated genes in breast cancer patients from BioXpress database whose expression is altered in the opposite direction huATN-658 + Zometa combination. **e** qPCR validation of selected genes (*HIST1H2AC, HIST1H2BD, SERPINE1, HIST1H2BK, EGR1*) obtained from RNA-Seq that were also found be altered in the opposite direction in breast cancer patients was done using the RNA from control and different treatment groups. Results are shown as mean ± SEM of samples obtained from at least three independent experiments. Statistical analyses were done using ANOVA and significant differences are represented by an asterisk (*P < 0.05)

To determine the global changes in gene expression, we compared the transcriptome of control IgG-treated MDA-MB-231 cells with that of huATN-658, Zometa, and huATN-658 + Zometa-treated cells by RNA-Seq using the Illumina NextSeq 500 Sequencing System (*n* = 3/group). Differential gene expression analysis was done by using Cufflinks as described in the ‘Methods and Materials’ section. A *P* value threshold of 0.05 was used to filter the significantly differentially expressed genes (DEGs) in each treatment group relative to the control. Our data showed that huATN-658 and Zometa monotherapy induced the differential expression of 61 (25 upregulated and 36 downregulated) and 222 (166 upregulated and 56 downregulated) genes, respectively (Fig. 6a, b, Supplementary File 2), when compared with IgG control-treated cells. In the huATN-658 + Zometa combination, 115 genes (67 upregulated and 48 downregulated) were differentially expressed in comparison with the control (Fig. 6a, b, Supplementary File 2). The top ten upregulated and top ten downregulated genes in each treatment group are shown separately in Supplementary File 1, Fig. S5.

The common and unique DEGs from different treatment groups are depicted by the Venn diagram (Supplementary File 1, Fig. S6). We found that only two genes (*UCA1*, *CCDC146*) were common in all three treatment groups. The huATN-658 + Zometa combination showed 7 and 66 common DEGs with huATN-658 and Zometa single agent treated groups, respectively. Moreover, the combination treatment uniquely changed the expression of 44 genes (listed in Supplementary File 1, Fig. S6) that were not differentially regulated by either one of the monotherapies. Gene ontology analysis revealed that the DEGs from each treatment group take part in a wide variety of cellular and biological processes that have a diverse molecular function (Supplementary File 1, Fig. S7). The top hits in the list of ‘molecular functions’ for all three treatment groups included ‘protein binding’ and ‘ion binding’.

Next, we performed a pathway analysis using the DEGs from huATN-658, Zometa, and huATN-658 + Zometa-treated cells to gain insights about the functional pathways affected after the different treatments (Fig. 6c). The top signaling pathway that showed the highest enrichment after huATN-658 treatment was ‘Signaling by transforming growth factor-beta (TGF-β) Receptor Complex’ while the top enriched pathway for both Zometa alone and huATN-658 + Zometa combination-treated groups was ‘Extracellular matrix organization’. Further analysis of all the enriched pathways upon huATN-658 + Zometa treatment revealed that genes from several crucial pathways related to cancer (for example, ‘Signaling by Nuclear Receptors’; ‘Regulation of nuclear SMAD2/3 signaling’; ‘Beta3 integrin cell surface interactions’; ‘Formation of the beta-catenin: TCF transactivating complex’) and bone remodeling (for example, ‘Transcriptional regulation by RUNX1′; ‘AP-1 transcription factor network’) are differentially regulated (Supplementary File 1, Table S1).

We then assessed the overlap between the DEGs induced by huATN-658 + Zometa treatment with the list of genes that are differentially expressed in breast cancer patients by using the BioXpress database.^32^ Our data showed that the combination treatment downregulated 14 genes that are typically overexpressed in breast tumors (Fig. 6d). In contrast, 22 genes that showed downregulation in cancer patients are upregulated by huATN-658 + Zometa treatment. We then validated the expression of some of the overlapped genes (*SERPINE1, HIST1H2BD, HIST1H2BK, HIST1H2AC, EGR1*) from Fig. 6d by quantitative real-time PCR (qPCR) (Fig. 6e). Finally, Kaplan–Meier analysis revealed that elevated expression *SERPINE1* and *HIST1H2BK* is associated with poor distant metastasis-free survival in patients with breast cancer (Supplementary File 1, Fig. S8). Taken together, our data indicate that the combination of huATN-658 + Zometa can regulate genes that impact metastatic breast cancer progression and survival.

## Discussion

Targeting of uPAR in general and specifically by the clinical candidate huATN-658 has shown significant anticancer effects in a number of preclinical studies^22,23^; Using huATN-658, we assessed the therapeutic potential for targeting breast cancer and for the first time, breast tumor-induced bone disease. We showed that huATN-658 effectively reduced MDA-MB-231 mammary tumor growth. Database analyses showed that the expression of the gene encoding uPAR (*PLAUR*) is observed in the luminal, HER2+, and triple-negative subtypes of breast cancer, and that expression is higher in the subtypes where metastatic recurrence is fastest. This in-depth analysis is highly significant in developing a therapeutic approach for patients with breast cancer and may be of particular utility for clinical development in patients with TNBC, where the first targeted therapy (Atezolizumab) was only recently approved. In addition, the ability of huATN-658 to block skeletal lesion further proteinates its clinical impact and broadens the targeted patient population.^22,24^

The majority of new cancer therapeutics being evaluated in preclinical studies at present are directed towards reducing tumor volume and visceral metastases but are rarely tested for their effect on non-visceral metastatic sites such as the skeleton. The skeleton is one of the major sites where breast tumor cells migrate, seed, and continue to grow to affect bone remodeling but is under-represented in the development of new drugs targeting breast cancer metastasis. Apart from causing a marked decline in the overall QOL, bone metastasis may also cause death related to skeletal-related complications.^33^ Radiotherapy as well as systemic treatments using different chemotherapeutic and endocrine agents are commonly used for the treatment of bone metastasis.^33,34^ Moreover, agents such as the bisphosphonates (e.g., Zometa) and antibodies against the receptor activator of nuclear factor kappa-B ligand (Denosumab) are also used for patients with skeletal metastasis.^35^ However, most of the currently available treatment strategies are used to palliate against skeletal fractures arising from breast cancer metastatic lesions and have little effect on metastatic progression. Therefore, there is an unmet need for novel therapeutic strategies to block breast tumor cell growth within the bone microenvironment.

Skeletal metastasis is a multistep event regulated and driven by the interaction between cancer cells and the bone microenvironment that acts as a vicious cycle dysregulating the normal remodeling of bone while increasing the propensity of tumor cell growth within the bone microenvironment.^36^ A recent study demonstrated that uPAR expression increases during osteoclast formation, and knockdown of the gene encoding for uPAR completely inhibits the formation of osteoclasts.^37^ Therefore, in this study, we evaluated the potential additive benefit of combination therapy with huATN-658 and Zometa that would be able to target both tumor growth and the osteolytic sequelae of breast cancer-induced bone disease. We chose to use the third-generation bisphosphonate Zometa in this study due to its higher half-life in bone (10 years)^38^ and cost-effectiveness because of its availability as a generic drug. In clinical settings, breast cancer patients with bone metastasis are predominantly of the osteolytic variety due to increased production of bone-resorbing growth factors and cytokines.^39^ The highly aggressive MDA-MB-231 and the bone metastatic MDA-BoM-1833 breast cancer cell lines represent the TNBC subtype and form osteolytic lesions when injected into the intratibial region of rodent models, providing a unique opportunity to study breast tumor-induced skeletal lesion.^40^ Although skeletal metastasis occurs less frequently with TNBC than other breast cancer subtypes, the MDA-MB-231 model mimics both skeletal breast cancer metastasis sequelae as well as growth and allows for simultaneous assessment of therapeutic effects on tumor and bone growth. Our study showed that the huATN-658 + Zometa combination treatment significantly reduced the skeletal lesions in immunocompromised mice without affecting the growth plate of the bone (Fig. 4b). Radiological and histological analysis of skeletal lesions in the combination-treated animals also showed that the structure of bone remained intact in most of the animals treated with combination therapy. In addition, there was a significant decrease in the number of osteoclasts in the huATN-658 + Zometa combination cohort relative to the control animals as shown by the TRAP staining in Fig. 5a to demonstrate that decreased osteolysis following these treatments has a net increase in bone volume. Since uPAR is also involved in osteoclast formation,^37^ the addition of huATN-658 in the combination cohort further inhibits osteoclastogenesis. Moreover, the RNA-Seq results showed that the huATN-658 + Zometa combination treatment significantly increases the expression of *COL1A2* and *SPOCK3* genes encoding for type 1 collagen and osteonectin proteins. These proteins are implicated in bone remodeling.^41^ The intratibial model used in this study may not be ideal for studying skeletal metastasis; however, it adequately assesses tumor cells colonization and growth in the skeleton and may not fully assess the antimetastatic potential. However, from the in vitro studies on a panel of breast cancer cell lines, we have shown that the invasiveness of the cells is significantly reduced upon huATN-658 + Zometa combination therapy, which suggests that proposed strategy will be useful in reducing the breast cancer metastasis in vivo.

Even though huATN-658 monotherapy effectively attenuated primary mammary tumor growth and skeletal lesion following mammary fat pad and intratibial inoculation, the magnitude of the anticancer effects seen in vitro was lower than that seen in vivo. These findings are consistent with previous studies in prostate and colorectal cancer.^22,42^ This discrepancy can be attributed to the complex nature of the tumor microenvironment, which is far more complex than 2D monolayers in vitro and huATN-658 may also change the expression of growth factors and cytokines affecting the tumor microenvironment.^42^ All these events may potentiate the further decrease in cell proliferation as seen in vivo. It should also be noted that huATN-658 only binds to uPAR on the xenografted tumor and not on the murine host cells.^24^ Therefore, xenograft models have the limitations of not demonstrating the full complement of effects of huATN-658 that might be observed upon its binding to host uPAR on immune and tumor microenvironment cells. Future clinical trials on human patients would be able to demonstrate the full complement of effects of huATN-658.

**Fig. 7:**
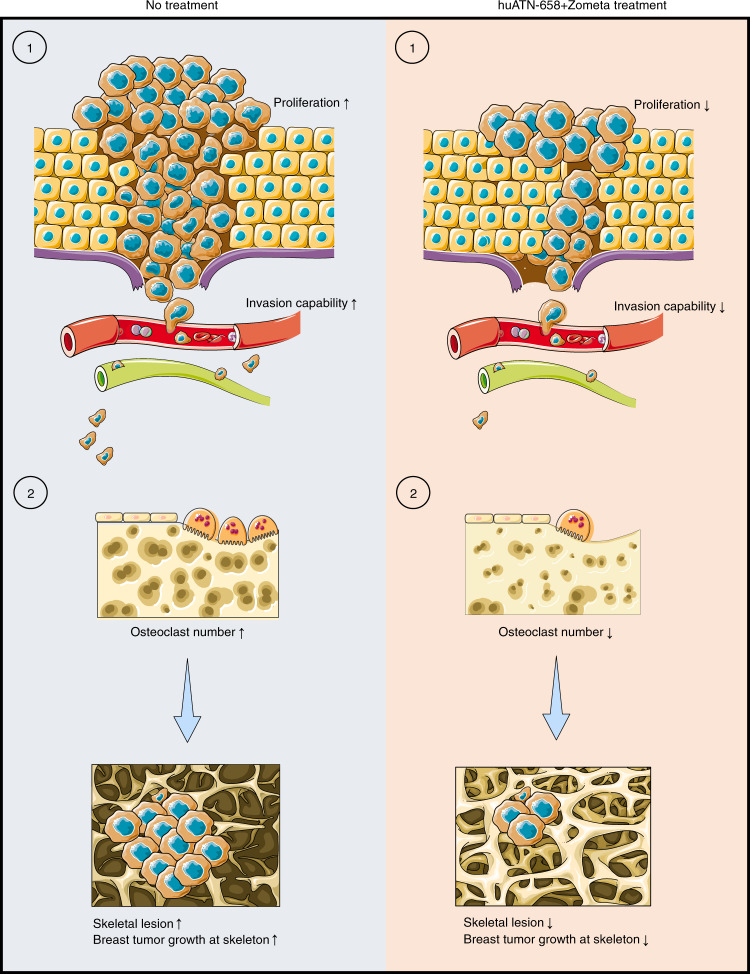
Summary of the anticancer effect of huATN-658 + Zometa combination. (1) The combination treatment reduces tumor cell proliferation and invasion in vitro, which suggests that such treatment strategy may also reduce tumor cell growth and metastasis to different organ in vivo. As shown in this study, the huATN-658 monotherapy significantly reduces mammary tumor volume in immunocompromised mice in vivo. However, further studies are warranted to assess whether huATN-658 in combination with Zometa treatment could reduce primary tumor volume and metastasis using appropriate models. (2) In vivo evidence obtained from intratibial injection of breast cancer cells revealed that the combination treatment reduces tumor cell growth and skeletal lesion. In addition, the combination treatment reduces the number of osteoclasts, which may also help to decrease the skeletal lesions

In summary, we have provided evidence that targeting uPAR with our clinical candidate huATN-658 is a viable and effective approach for treating breast cancer. Moreover, the combination of huATN-658 + Zometa not only protects against the formation of skeletal lesions induced by metastatic breast cancer cells but also blocks further tumor growth within the bone microenvironment (Fig. 7). Consistent with these observations, combination treatment reduced the expression of genes implicated in the TGF-β signaling pathways while promoting the genes involved in osteoblast formation. Taken together, results from these studies support assessing huATN-658 in combination with Zometa in women with breast cancer-associated skeletal metastasis as a clinical development strategy.

## Materials and methods

### Database analyses

The differential gene expression pattern of *PLAUR* was assessed in 31 different cancer types using the GEPIA2^43^ tool through the comparison TCGA tumor samples with the paired adjacent normal samples from TCGA and Genotype-Tissue Expression (GTEx) database. Further, validation of the differential expression of *PLAUR* was done using publicly available Oncomine^44^ database that includes both microarray and RNA-Sequencing data from different studies. The expression of *PLAUR* in different subtypes and stages of breast cancer was assessed by comparing normal vs. tumor tissues using UALCAN^45^ portal. The prognostic significance of the *PLAUR* gene in breast cancer was assessed using the microarray-based gene expression data from 1 746 breast cancer patients from the KM-plotter database.^46^ The expression of *PLAUR* and *PLAU* genes in 51 different breast cancer cells was analyzed via the selection of Neve et al. database in Gene Expression-Based Outcome for Breast Cancer (GOBO) webserver.^47,48^

### Cell culture and treatments

Human MDA-MB-231 and Hs578T breast cancer cells (ATCC; Manassas, Virginia) were cultured and maintained as previously described by us.^28^ The authentication of these two cell lines was done at the Genetic Analysis Facility (SickKids, Toronto, Canada). The parental bone metastatic MDA-BoM-1833 cell line was a kind gift from Dr Joan Massagué (Memorial Sloan-Kettering Cancer Center, NY, USA). For all in vitro experiments, cells were treated for 2 days (48 h) with 50 μg·mL^−1^ huATN-658 (Attenuon, LLC; San Diego, CA, USA), 10 μmol·L^−1^ Zometa (Novartis, Basel, Switzerland), or their combination by direct addition into the culture medium. The control cells were treated with isotype-matched IgG antibody.

### Cell proliferation and invasion assay

For cell proliferation assay, MDA-MB-231, Hs578T, and MDA-BoM-1833 cells were plated onto each well of six-well plates and treated with IgG control (50 μg·mL^−1^), huATN-658 (50 μg·mL^−1^), Zometa (10 μmol·L^−1^), and huATN-658 + Zometa for 2 days (48 h). At the end of the experiment, trypsinized cells were counted by using a Coulter counter (Model ZF; Coulter Electronics, Hertfordshire, UK). The following equation was used to determine the CDI, CDI = AB/(A × B).^49,50^ In this case, ‘AB’ indicates cell growth in combination treatment relative to the control cells while ‘A’ and ‘B’ indicate cell growth upon monotherapy treatment relative to the control. A CDI value less than 1 indicates a synergistic effect of the combination, CDI value of 1 indicates an additive effect while a CDI value greater than 1 indicates an antagonistic effect. The Boyden chamber Matrigel invasion assay, that determines the invasiveness of the tumor cells, was carried out as described by us before.^28^

### Animal protocols

All the animal studies done are in full compliance with protocols that are approved by the McGill University Facility Animal Care Committee. For the studies related to the orthotopic model of breast cancer, logarithmically growing viable MDA-MB-231 breast tumor cells at the density of 5 × 10^5^ cells mixed with Matrigel in 200 μL phosphate-buffered saline (PBS) were inoculated into the fourth mammary fat pad of 4–6-week-old female CD-1 nude mice (Charles River Laboratories, St. Constant, Québec, Canada). The animals were monitored for the emergence of a measurable tumor. When the average tumor volume reached 50–100 mm^3^, they were randomized into three groups: PBS as vehicle alone, 10.0 mg·kg^−1^ human isotype-matched IgG, or huATN-658 antibody twice a week via intraperitoneal route for 5 weeks. Tumor volumes were measured using a caliper and calculated by the following formula: *V* = (length × Width^2^)/2. For the intratibial model of breast cancer, 2 × 10^5^ MDA-MB-231 or 5 × 10^4^ MDA-BoM-1833 cells were injected into the tibial region of CD-1 nude mice. At day 3 following intratibial injection of these cells, immunocompromised CD-1 mice were treated via intraperitoneal route with control IgG, huATN-658 antibody (10 mg·kg^−1^) twice per week (2QW), Zometa (100 µg·kg^−1^) once per week (QW), or huATN-658 + Zometa combination until experimental endpoint which was set at 10 and 6 weeks for MDA-MB-231 and MDA-BoM-1833 cell injected CD-1 mice, respectively.

### Radiological and immunohistochemical analyses

At weeks 4,6, and 10 post intratibial inoculation of MDA-MB-231 cells, the animals were anesthetized, and the hindlimbs were radiographically assessed for skeletal lesions by Kubtec digital X-ray (Kubtec Medical Imaging, Stratford, CT, USA). The skeletal lesions were quantified using Image J (Fiji plugin).

At the end of these studies on week 10, all animals were sacrificed, left tibias were fixed in periodate-lysine-paraformaldehyde solution, decalcified, embedded in paraffin and subjected to H&E staining. Tumor area was determined using Image J (Fiji plugin). Tibia from control and experimental groups of animals were subjected to TRAP to measure the number of osteoclasts (Sigma Aldrich, Oakville, Ontario, Canada) and immunohistochemical assessment of the fixed tissues was done using an antibody against cell proliferation marker Ki67 (Cat# M7240, Dako, Glostrup, Denmark) at the histopathology platform of RI-MUHC, Montréal, QC, Canada. The percentage of Ki67 positive tumor cells from randomly selected fields were counted by an automated approach using the color deconvolution algorithm of ‘ImmunoRatio’ application.^51^ For the animals injected with MDA-BoM-1833 cells, a radiological assessment was done by Bruker In-Vivo Xtreme imaging system at week 6 post intratibial inoculation.

### RNA-Seq and analysis pipeline

Total RNA from biological replicates obtained from three independent experiments were isolated using the AllPrep DNA/RNA Mini Kit (Cat# 80204, Qiagen, Hilden, Germany). There were four groups: control IgG, huATN-658, Zometa, and huATN-658+Zometa, all of which passed by quality control by the Agilent 2100 Bioanalyzer. Following that, samples were prepared following the standard protocol for the NEBnext Ultra ii Stranded mRNA kit (New England Biolabs, Massachusetts, United States), and paired-end sequencing was done on the Illumina NextSeq 500 System. After completion of the sequencing run, the de-multiplexed read sequences were aligned to the *Homo sapiens* hg19 reference sequence using STAR aligners.^52^ Assembly and differential expression were estimated using the Cufflinks.^53^ The pathway analyses of the DEGs in different treatment groups were carried out by using the publicly available ConsensusPathDB.^54^

We also validated the expression of several genes by qPCR using the primers listed in Supplementary File 1, Table S2. The qPCR assay was performed following as described by us previously.^55^ The changes in gene expression in different treatment groups relative to the control samples were determined by the 2^−ΔΔC^^t^ method.

### Statistical analysis

Results are shown as the mean ± standard error of the mean (SEM). Statistical significance was determined by Student’s *t* test, ANOVA followed by Tukey’s post hoc tests as appropriate. The *P* value cutoff was set at less than 5% to be considered as statistically significant.

## Supplementary information

Supplementary File 1

Supplementary File 2

## Data Availability

The authors of the paper declare that all relevant data generated or analyzed during this study are available within the main article and the supplementary files.
