# Supplementary material for: uPAR antibody (huATN-658) and Zometa reduce breast cancer growth and skeletal lesions
Source: Bone Res. 2020 Apr 17;8:18. doi: 10.1038/s41413-020-0094-3 (PMC7165173; doi:10.1038/s41413-020-0094-3)
Supplement: Supplementary file 1 [file 41413_2020_94_MOESM1_ESM.pdf]

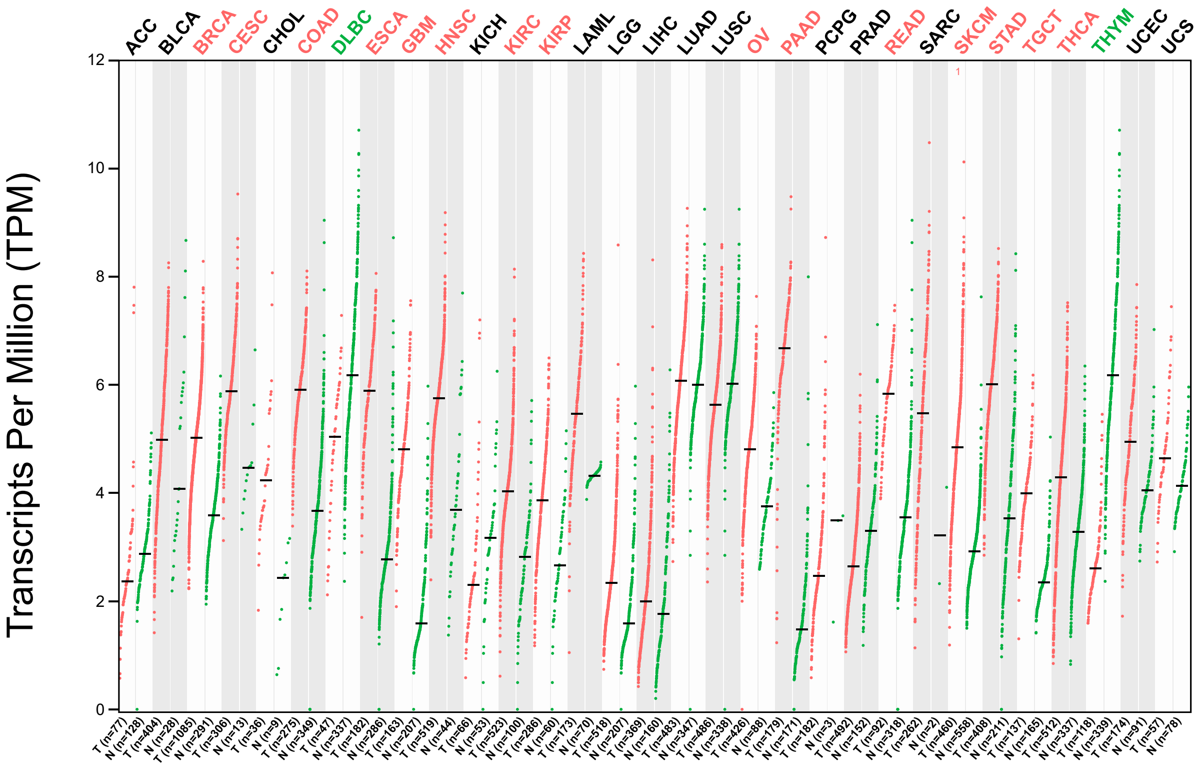

Figure S1: Dot plot of *PLAUR* expression in different cancer types ('red' and 'green' dots are for tumors and normal tissues, respectively). The significantly upregulated or downregulated *PLAUR* expression values in tumors vs. normal tissue samples are shown by respective 'red' and 'green' font of cancer types abbreviations on top of the graph. The cancer types where there is no change in *PLAUR* expression values between tumors and adjacent normal tissue samples are shown in 'black' font. The statistical analysis was done by ANOVA (|Log2FoldChange| =1 and q-value threshold of 0.01) using the GEPIA2 tool. The number of the tumor (T) and normal (N) tissue samples used for each cancer types are shown at the bottom of the graph.

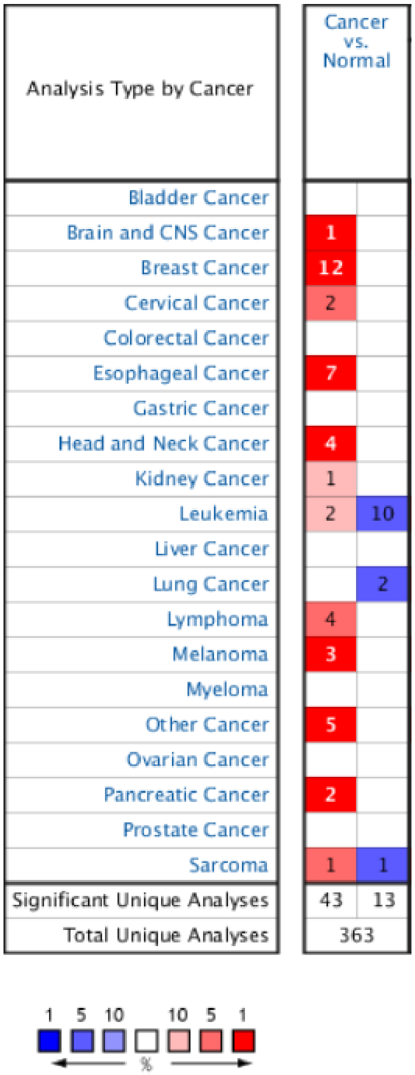

Figure S2: *PLAUR* expression in 20 different cancer types from the Oncomine database. Differential gene expression is shown by different colors where 'red' color represents upregulation and 'blue' color represents downregulation of the *PLAUR* gene. The number within each of the squares indicate the frequencies of analysis that satisfy the following threshold: *P*-value=10^{-4}, fold change= 2, and gene rank percentile=10%. The expression of *PLAUR* in breast cancer is significantly elevated according to Oncomine.

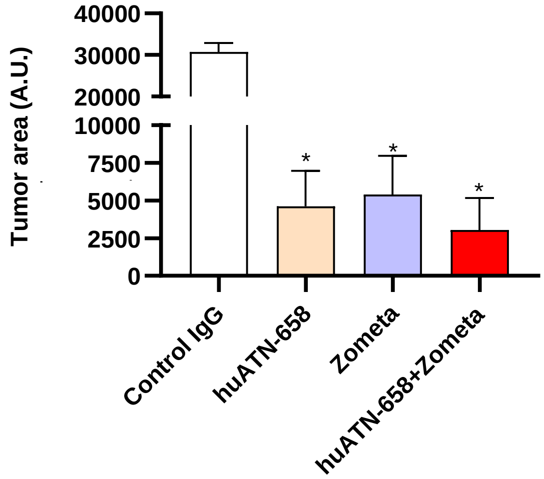

Figure S3: Bar graph of the average tumor areas in the H&E stained tumors from control and different treatment groups as determined by ImageJ (Fiji plugin). Significant differences were determined using ANOVA followed by *post hoc* Tukey's test and are represented by asterisks. (n=7/group)

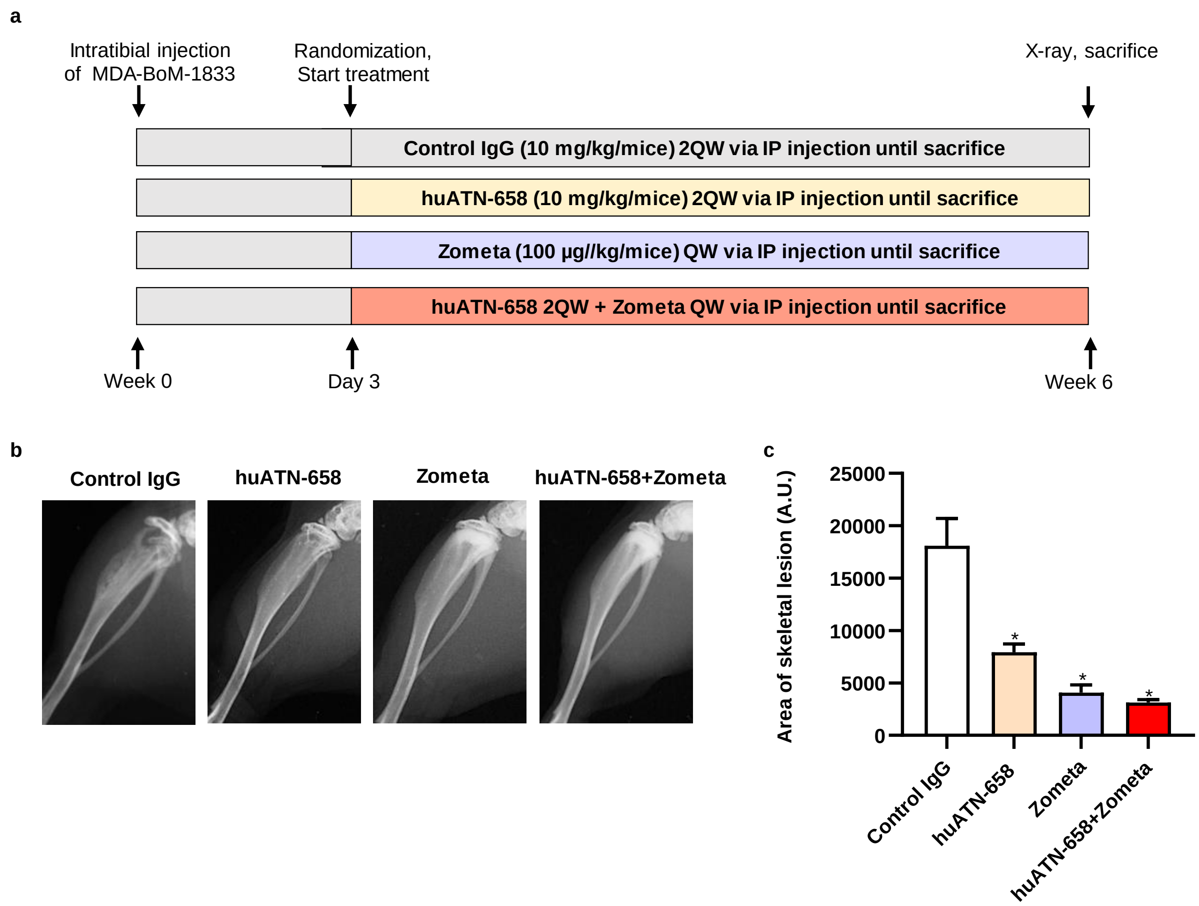

Figure S4: Effect of huATN-658 and Zometa on MDA-BoM-1833 skeletal lesions. a Schematic of the protocol used for treatment with huATN-658, Zometa and their combination. **b** Representative X-rays of the tibia used from each treatment group is shown. **c** Lesion area was determined by ImageJ and plotted as bar graph. The error bars indicate mean ± SEM. Significant differences in skeletal lesions from the control IgG animals were determined using ANOVA followed by *post hoc* Tukey's test and are represented by an asterisk (*$P<0.05$).

huATN-658 vs Control IgG

Gene	log2(Fold change)	*P*- value
HIST2H2BC	-2.04714	0.0292
EEF1DP3	-2.0338	0.02295
FOLR1	-1.21985	0.01695
MAMSTR	-1.12608	0.0067
CAPN8	-1.11257	0.004
LINC01088	-1.06847	0.01945
GADD45G	-1.03011	0.0306
ZNF826P	-1.02046	0.03415
SUN3	-0.941588	0.02045
ATP6V1B1-AS1	-0.9218	0.02
C4orf27	0.435366	0.0176
PRELID2	0.440775	0.04295
ZNF181	0.462776	0.049
TMEM128	0.468583	0.02325
ICA1	0.522992	0.03475
FAM185A	0.59089	0.0357
CCDC146	0.630723	0.04655
VIPR1	0.684805	0.02765
OVCH2	0.739521	0.04255
PRG2	0.872198	0.0409

Zom vs Control IgG

Gene	log2(Fold change)	*P*- value
CD38	-1.0667	0.02615
AKR1E2	-0.878674	0.0288
RIMS2	-0.836104	0.00315
TMEM74	-0.767033	0.03745
ADAMTS6	-0.715249	0.0099
HIST1H2BD	-0.712599	0.00335
AMIGO1	-0.684077	0.04095
HIST1H2AC	-0.62198	0.00855
WDR4	-0.567342	0.0303
KCNQ3	-0.567087	0.0227
CLPSL2	1.97534	0.0112
MIR210HG	1.794	0.00005
LOC101928674	1.78988	0.02985
RHOB	1.42929	0.00005
SMIM2-AS1	1.11306	0.0441
ZNF575	1.0853	0.0191
ALDOC	1.06738	0.00005
PI3	1.04928	0.01535
PECAM1	1.01007	0.00055
LOC101926941	1.00825	0.0236

huATN-658+Zom vs Control IgG

Gene	log2(Fold change)	*P*- value
RPL21	-1.85453	0.0014
TP53TG1	-1.3104	0.02165
C4orf22	-1.24005	0.03415
FOSB	-1.04025	0.00135
ATP6V1B1-AS1	-1.00263	0.01455
LINC00632	-0.941262	0.04975
PPFIBP2	-0.822877	0.01695
ID2	-0.709885	0.02795
MAMDC2	-0.702318	0.0034
ZNF879	-0.698864	0.0235
RIBC2	0.840532	0.019
CCDC146	0.879768	0.00595
MUC5AC	0.884492	0.0001
LOC101926941	0.920203	0.0431
UBE2E4P	0.973199	0.029
C1S	0.975045	0.0064
RHOB	1.0208	0.00005
MIR210HG	1.31691	0.00035
CLPSL2	1.59546	0.03105
COL1A2	1.69531	0.00005

Figure S5: Heatmap of top 10 upregulated and 10 downregulated genes (based on fold change) that are differentially expressed in each treatment groups compared to control. The upregulated and downregulated genes are shown by ‘red’ and ‘blue’ color filled boxes, respectively.

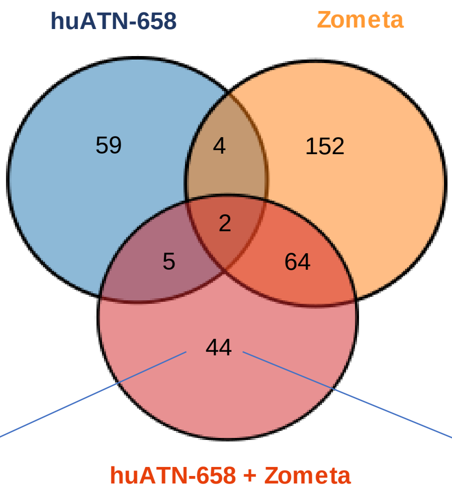

RPL21, TP53TG1, C4orf22, LINC00632, ID2, MAMDC2, ZNF879, ZNF174,LRRC2, FAM222A, DMKN, ALPP, ATP1A3, SLAMF7, RAD54L2, FOXK1, PARD3B, NOL9, SH2D5, BDNF, GPRC5A, MYBBP1A, DHX37, CDC42EP1, SERPINE1, CREBBP, C15orf48, FAM20C, ANKRD9, FAM210B, ARHGAP9, ANKFN1, CBR3, RGCC, FBLN5, NUDT8, CYP2E1, LRAT, CAPS2, SPOCK3, CTSK, UBE2E4P, C1S, COL1A2

Figure S6: Venn diagram showing the commonalities and uniqueness of different treatment at the level of gene expression. The 44 genes uniquely altered by huATN-658+Zometa combination are listed in the bottom. The huATN-658+Zometa combination had 7 (5+2) and 66 (64+2) common DEGs with huATN-658 and Zometa monotherapy treated groups respectively.

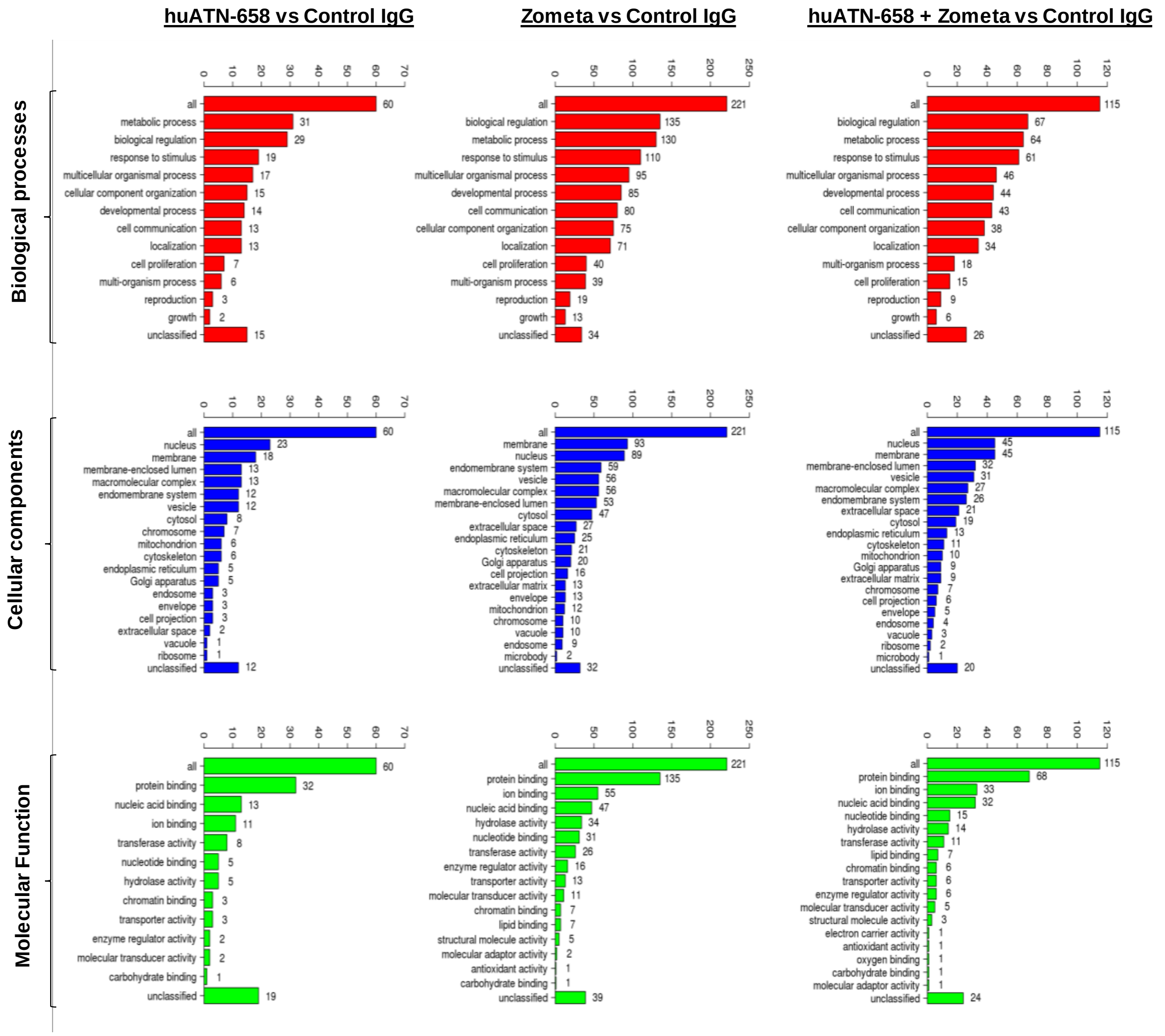

Figure S7: DEGs from each treatment groups were analyzed for Gene ontology (GO) enrichment (GOSlim) using Webgstalt[1]. For huATN-658 treated group, 60 out of the 61 DEGs were analyzed. For Zometa treated group, 221 out of 222 DEGs were analyzed by GOSlim while all 115 DEGs from the huATN-658+Zometa-treated groups were analyzed for GO enrichment.

Table S1: Complete list of pathways that are regulated by the differentially expressed genes from huATN-658+Zometa combination treatment

**Pathways regulated by huATN-658+Zometa**	**Target Genes**
Extracellular matrix organization	*SERPINE1; FBLN5; PECAM1; CTSK; ADAMTS1; ITGB8; THBS1; COL1A2; LOX; SPOCK3*
Estrogen-dependent gene expression	*FOSB; HIST1H2BD; HIST1H2AC; CXXC5; CITED1; CREBBP; HIST1H2BK*
ESR-mediated signaling	*FOSB; HIST1H2BD; HIST1H2AC; CXXC5; CITED1; CREBBP; HIST1H2BK*
Glycolysis / Gluconeogenesis	*ALDH3A2; ACSS2; ALDOC; ALDH3A1; ENO2*
Signaling by Nuclear Receptors	*FOSB; HIST1H2BD; HIST1H2AC; CXXC5; CITED1; CREBBP; HIST1H2BK*
mTOR signaling pathway	*ULK1; DDIT4; PPARGC1A; BNIP3*
HIF-1-alpha transcription factor network	*ID2; SERPINE1; CREBBP; BNIP3*
Circadian Clock	*NOCT; SERPINE1; PPARGC1A*
Regulation of nuclear SMAD2/3 signaling	*CITED1; COL1A2; SERPINE1; CREBBP*
Transcriptional regulation by RUNX1	*CTSK; THBS1; HIST1H2BD; CREBBP; HIST1H2AC; HIST1H2BK*
Complement and coagulation cascades	*C1S; F3; CFB; SERPINE1*
Amyloid fiber formation	*HIST1H2BD; HIST1H2AC; CALB1; HIST1H2BK*
Signaling events mediated by HDAC Class III	*ACSS2; PPARGC1A; CREBBP*
Direct p53 effectors	*DDIT4; BNIP3L; SERPINE1; CREBBP; RGCC*
C-MYB transcription factor network	*PTGS2; COL1A2; CREBBP; ZFHX3*
Activation of the TFAP2 (AP-2) family of transcription factors	*CITED1; CREBBP*
VLDLR internalisation and degradation	*PCSK9; VLDLR*
Beta3 integrin cell surface interactions	*PECAM1; COL1A2; THBS1*
RUNX1 regulates genes involved in megakaryocyte differentiation and platelet function	*HIST1H2BD; THBS1; HIST1H2AC; HIST1H2BK*
Formation of the beta-catenin:TCF transactivating complex	*HIST1H2BD; HIST1H2AC; CREBBP; HIST1H2BK*
B-WICH complex positively regulates rRNA expression	*HIST1H2BD; MYBBP1A; HIST1H2AC; HIST1H2BK*
BMAL1:CLOCK,NPAS2 activates circadian gene expression	*NOCT; SERPINE1*
RHO GTPases activate PKNs	*HIST1H2BD; RHOB; HIST1H2AC; HIST1H2BK*
AGE-RAGE signaling pathway in diabetic complications	*F3; COL1A2; EGR1; SERPINE1*
Regulation of Androgen receptor activity	*EGR1; CREBBP; SPDEF*

Packaging Of Telomere Ends	*HIST1H2BD; HIST1H2AC; HIST1H2BK*
Phase I - Functionalization of compounds	*ACSS2; ALDH3A1; CYP2E1; CBR3*
Positive epigenetic regulation of rRNA expression	*HIST1H2BD; MYBBP1A; HIST1H2AC; HIST1H2BK*
Biosynthesis of DHA-derived SPMs	*PTGS2; CYP2E1*
Human papillomavirus infection	*PTGS2; ITGB8; OASL; THBS1; COL1A2; LFNG; CREBBP*
Alcoholism	*HIST1H2BD; BDNF; FOSB; HIST1H2AC; HIST1H2BK*
Biosynthesis of specialized proresolving mediators (SPMs)	*PTGS2; CYP2E1*
TCF dependent signaling in response to WNT	*HIST1H2BD; HIST1H2AC; CREBBP; SOX4; HIST1H2BK*
TP53 Regulates Transcription of Genes Involved in Cytochrome C Release	*BNIP3L; CREBBP*
Arachidonic acid metabolism	*PTGS2; CBR3; CYP2E1*
RNA Polymerase I Promoter Opening	*HIST1H2BD; HIST1H2AC; HIST1H2BK*
Meiotic recombination	*HIST1H2BD; HIST1H2AC; HIST1H2BK*
Mitophagy	*ULK1; BNIP3L; BNIP3*
DNA methylation	*HIST1H2BD; HIST1H2AC; HIST1H2BK*
Integrin cell surface interactions	*ITGB8; PECAM1; THBS1*
Activated PKN1 stimulates transcription of AR regulated genes KLK2 and KLK3	*HIST1H2BD; HIST1H2AC; HIST1H2BK*
Histidine metabolism	*ALDH3A2; ALDH3A1*
FoxO signaling pathway	*CCNG2; KLF2; CREBBP; BNIP3*
SIRT1 negatively regulates rRNA expression	*HIST1H2BD; HIST1H2AC; HIST1H2BK*
Systemic lupus erythematosus	*C1S; HIST1H2BD; HIST1H2AC; HIST1H2BK*
AP-1 transcription factor network	*FOSB; EGR1; COL1A2*

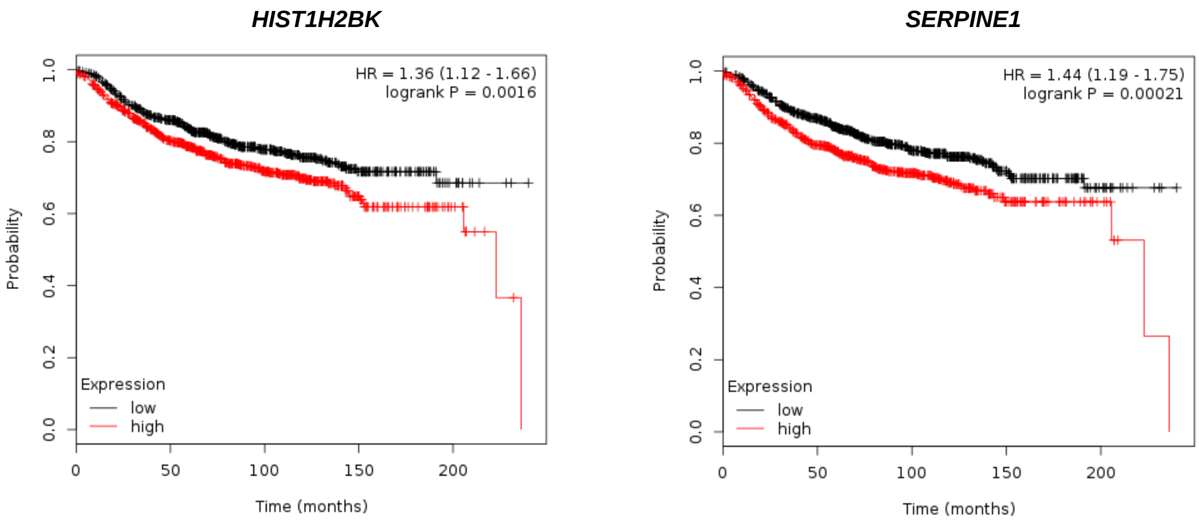

Figure S8: Kaplan-Meier plot of distant metastasis free survival (DMFS) using a dataset of breast cancer patients from the KM-plotter database[2] revealed that lower expression of *HIST1H2BK* and *SERPINE1* are associated with increased survival probabilities.

Table S2: Primers used in this study are listed below[3-6]

**Gene Name**		**Sequences used for qPCR (5'→ 3')**
SERPINE1	For	AGGAGGAGAAACCCAGCAGC
	Rev	GCCCTGGACCAGCTTCAGAT
HIST1H2BK	For	CACCAGCGCTAAGTAAACTTGCCA
	Rev	AGAGGCCAGCTTTAGCTTGTGGAA
HIST1H2BD	For	GATGCCTGAACCTACCAAGT
	Rev	GCTTCTTCCCGTCCTTCTTC
HIST1H2AC	For	GACGAGGAGCTCAACAAACTG
	Rev	ACCTGTCAAATCACTTGCCC
EGR1	For	AGCCCTACGAGCACCTGAC
	Rev	GGTTTGGCTGGGGTAACTG
GAPDH	For	TGCACCACCAACTGCTTA
	Rev	AGAGGCAGGGATGATGTTC

References

1 Wang, J., Vasaikar, S., Shi, Z., Greer, M. & Zhang, B. WebGestalt 2017: a more comprehensive, powerful, flexible and interactive gene set enrichment analysis toolkit. *Nucleic acids research* **45**, W130-W137, doi:10.1093/nar/gkx356 (2017).

2 Gyorffy, B. *et al.* An online survival analysis tool to rapidly assess the effect of 22,277 genes on breast cancer prognosis using microarray data of 1,809 patients. *Breast Cancer Res Treat* **123**, 725-731, doi:10.1007/s10549-009-0674-9 (2010).

3 Masuda, T. *et al.* Off Target, but Sequence-Specific, shRNA-Associated Trans-Activation of Promoter Reporters in Transient Transfection Assays. *PloS one* **11**, e0167867-e0167867, doi:10.1371/journal.pone.0167867 (2016).

4 Li, X. *et al.* Identification of a histone family gene signature for predicting the prognosis of cervical cancer patients. *Scientific Reports* **7**, 16495, doi:10.1038/s41598-017-16472-5 (2017).

5 Pirngruber, J. *et al.* CDK9 directs H2B monoubiquitination and controls replication-dependent histone mRNA 3'-end processing. *EMBO reports* **10**, 894-900, doi:10.1038/embor.2009.108 (2009).

6 Ponti, D. *et al.* The expression of B23 and EGR1 proteins is functionally linked in tumor cells under stress conditions. *BMC cell biology* **16**, 27-27, doi:10.1186/s12860-015-0073-5 (2015).
